# Sleep Quality and Performance in Professional Athletes Fasting during the Month of Ramadan

**DOI:** 10.3390/ijerph18136890

**Published:** 2021-06-27

**Authors:** Anna Lipert, Remigiusz Kozłowski, Paweł Rasmus, Michał Marczak, Małgorzata Timler, Dariusz Timler, Ewa Kaniecka, Abedelmajid Nasser, Mohammad Ghaddar, Ali Ghaddar

**Affiliations:** 1 Department of Sports Medicine, Medical University of Lodz, 92-213 Lodz, Poland; anna.lipert@umed.lodz.pl; 2 Center of Security Technologies in Logistics, Faculty of Management, University of Lodz, 90-237 Lodz, Poland; remigiusz.kozlowski@wz.uni.lodz.pl; 3 Department of Medical Psychology, Medical University of Lodz, 90-131 Lodz, Poland; pawel.rasmus@umed.lodz.pl; 4 Department of Management and Logistics in Healthcare, Medical University of Lodz, 90-131 Lodz, Poland; michal.marczak@umed.lodz.pl (M.M.); malgorzata.timler@stud.umed.lodz.pl (M.T.); 5 Department of Emergency Medicine and Disaster Medicine, Medical University of Lodz, 92-213 Lodz, Poland; dariusz.timler@umed.lodz.pl (D.T.); ewa.kaniecka@stud.umed.lodz.pl (E.K.); 6 Observatory of Public Policies and Health, Beirut 03-690, Lebanon; nasser.abedelmajid@gmail.com (A.N.); ghaddarmohammad@gmail.com (M.G.); 7 Department of Biomedical Sciences, Lebanese International University, Beirut 03-690, Lebanon

**Keywords:** fasting, physical performance, Ramadan, sleep

## Abstract

Background: Maintaining physical performance during Ramadan Diurnal Fasting (RDF) is a challenge for professional athletes. The literature shows that sleep disturbances experienced by athletes during RDF are associated with reduced physical performance. The effect of sleep quality on physical performance, and the effect of work status on physical performance during RDF among athletes, besides engaging in trainings, have been little investigated. This study aims to evaluate the effect of RDF on the physical performance of professional athletes taking into consideration their sleep quality and work status. Methods: Professional medium-distance male runners (n = 32) participated in our study in the summer of 2019. Data about socio-demographics, training characteristics, sleep quality (Pittsburg Sleep Quality Index: PSQI), physical performance (Cooper Test; Harvard step test) were collected before and during Ramadan. Student’s-test and Welch and Wilcoxon tests were used for data analysis. Results: Both quality of sleep and physical performance of athletes deteriorated during Ramadan. People with better quality of sleep had better physical fitness/performance both before and during RDF. Athletes who worked beside trainings achieved worse physical fitness test results and had worse quality of sleep. Conclusions: Policies aimed to improve physical performance in RDF should consider the quality of sleep and the work status of athletes.

## 1. Introduction

Athletes who adhere to the Islamic code practice fasting for a whole month during the year, the month of Ramadan. During Ramadan diurnal fasting (RDF), athletes completely refrain from eating and drinking from sunrise to sunset and usually eat one meal after sunset “iftar” and another before sunrise “souhour”. The changes in meal timing during Ramadan produce a reduction in the frequency of food and water intake and an increase in the frequency of consumption of high calorie food and drinks (heavy meals) [1,2]. Ramadan fasting decreases glucose level and influences weight reduction, what can result in a lack of glycogen in the muscle and muscle protein degradation [1]. It also influences Circardian rhythms [3,4,5] and produces modifications in the sleep cycle and sleep patterns, with significant sleep alteration and sleep disturbances including delays in wake-up time and bedtime, increases in subjective and objective daytime sleepiness, and a reduction in nocturnal sleep duration [6,7,8,9,10]. Moreover, it has been shown recently that RDF reduces total sleep time and increases excessive daytime sleepiness (Epworth sleepiness scale: ESS) [11].

Evidence about alternations in food intake and sleeping habits during RDF has led several researchers to explore the effect of RDF on athletes’ performance, though the evidence remains inconclusive. A recent systematic review concluded that RDF had no influence on the majority of physical performance parameters including strength, aerobic performance, jump height, fatigue and total work, though it negatively affected sprint performance [12]. In another study that controlled for food intake, sleeping time and training load, RDF appeared to reduce prolonged intermittent sprint performance in football players [13]. Zarrouk et al. concluded that RDF did not decrease neuromuscular performance, nor did it affect anthropometric parameters of athletes who engaged in RDF [14]. Similarly, other studies suggested that RDF had little or no effect on various performance tests among fasting athletes [15], and did not produce significant changes in physical performance in female athletes [16]. On the other hand, other research among football players suggested that RDF produces significant decrements in perceived and actual performance tests including speed, agility, dribbling speed, endurance [17], aerobic capacity and speed endurance performance [18]. Maughan & Shirreffs concluded that the dehydration associated with RDF could impair performance and recovery in competitions or events that last around one hour or more in athletes who engage in more than one event or training per day [19]. Similarly, it has been shown that dehydration in athletes increased heart rate and decreased ability to concentrate, reduced alertness and increased subjective sensations of fatigue [9].

While it has been clearly revealed that RDF increases the sensation of fatigue [20] and produces significant hormonal, metabolic, and inflammatory changes linked to sleep disturbances, changes in sleep patterns, reduction of hours of sleep, energy deficiency and fatigue in athletes [21], the topic has not yet been systematically evaluated. Further research is needed to deepen the understanding of sleep’s impact on performance [22]. Furthermore, the changes in the quality of sleep and its effect on athletes’ performance during RDF has been little investigated. In addition, the role of certain lifestyle-related variables, such as work status, that might influence physical performance of athletes during RDF has not been taken into consideration in previous research. We hypothesise that sleep quality decreases during RDF and reduces physical performance, and that athletes who have full-time job commitments besides engaging in trainings during RDF would have lower physical performance, independent on the quality of sleep. Therefore, the aim of the current study was to evaluate the effect of diurnal Ramadan fasting on the physical performance of professional athletes taking into consideration sleep quality and work status.

## 2. Material and Methods

### 2.1. Participants and Experimental Setting

The study was conducted during Ramadan month, 2019, with a length of fasting day of approximately 15 h. The average temperature and humidity were around 30 °C and 31% before Ramadan (BR) and 32 °C and 34% during Ramadan (DR). Data were collected at two time intervals (two weeks before Ramadan and during Ramadan (third week)), in accordance with other similar studies [12]. Participants were thirty-two healthy male athletes aged 17–40 years performing endurance training in the form of running, who voluntarily participated in the study. Athletes who had experienced transmeridian travel were not included in the study.

Participants were informed of details about the design of the study and the potential risks and benefits before providing their written informed consent. Participants were free to withdraw from the study at any time without further consequences. The study was approved by the Institutional Review Board of the Lebanese International University before the beginning of the assessments, approval Ref: LIUIRB--AG1).

### 2.2. Study Design

First, socio-demographic and training-related variables were collected using a self-made questionnaire. Then, in a prospective cohort design, data about sleep quality and physical performance were collected using The Pittsburg Sleep Quality Index (PSQI) and using the Cooper Test and the Harvard step test, respectively. All these measurements were repeated at two-time intervals, two-weeks before the start of Ramadan (baseline) and during 3rd week during Ramadan.

### 2.3. Data Collection

#### 2.3.1. The Pittsburgh Sleep Quality Index (PSQI)

The PSQI is self-report questionnaire and an effective instrument widely used by clinicians and researchers to broadly assess several dimensions of sleep and assess the general quality of sleep in adults. It differentiates “poor” from “good” sleep quality by measuring seven areas (components): subjective sleep quality, sleep latency, sleep duration, habitual sleep efficiency, sleep disturbances, use of sleeping medications and daytime dysfunction over the last month. A total score of five or greater is indicative of poor sleep quality. The PSQI has been validated in numerous populations and languages [23,24] as well as many medical populations such as patients with insomnia, patients with traumatic brain injury, patients with cancer, patients receiving bone marrow and renal transplants, nursing home residents and pregnant women [25,26]. The Arabic version had been validated and has demonstrated acceptable reliability (Cronbach’s alpha coefficient = 0.77) [27].

#### 2.3.2. The Cooper Test

The Cooper Test is a 12-min run test to determine aerobic fitness and provides an estimate of VO2max. The Cooper 12-min run test [28] requires the person being tested to run or walk as far as possible in a 12 min period. The objective of the test is to measure the maximum distance covered by the individual during the 12 min period and is usually carried out on a running truck by placing cones at various distances to enable measurement of the distance. The calculation of the estimated VO2 max results (in ml/kg/min) was performed according to the formula: VO2max = (22.351 × kilometers) − 11.288. There is a very high correlation between the distance ran (or walked) in 12 min and VO2 max value, which measures the efficiency of oxygen use while exercising.

#### 2.3.3. The Harvard Step Test

The Harvard step test is a type of cardiovascular endurance test that measures the efficiency of an athlete’s circulatory system and respiratory system in supplying oxygen to the working muscles and supporting sustained physical activity. It is also a good measurement of fitness and the ability to recover after strenuous exercise by checking the recovery rate (time needed for the heart rate to return to its normal rhythm). The test computes the capability of the individual to exercise continuously for extended intervals of time without tiring. Blood pressure was measured with a standardized upper arm cuff methodology using a “Reister” sphygmomanometer. After resting seated for 5 min, participants had three measurements taken from their left arm, with the average calculated. If there was a difference of ±5 mm Hg between the readings, researchers took an additional measurement.

### 2.4. Statistical Analysis

Statistical analyses were performed using Statistical version 13.1 software (StatSoft). When the Shapiro-Wilk-test revealed that data were normally distributed, parametric tests were performed and the statistical analysis was performed by Student’s t-test. When data were not normally distributed, nonparametric tests were used. The statistical analysis was performed by the Welch test for dependent variables or the Wilcoxon test for independent variables. Significant differences were accepted for all tests with *p* < 0.05.

## 3. Results

### 3.1. Characteristic of Participants

The characteristics of the study participants are shown in Table 1. Participants were around 28 ± 6.7 years old and had been practicing professional sports for around 10 ± 5 years. Almost half were working full time (44%) and the rest were either working part-time or students (56%). The average training hours per week was 10.59 ± 2.79 before Ramadan and 8.03 ± 1.66 during Ramadan. Almost half trained twice per day (44%) before Ramadan while only 0.03% trained twice during Ramadan.

### 3.2. Sleep Quality

**Figure 1:**
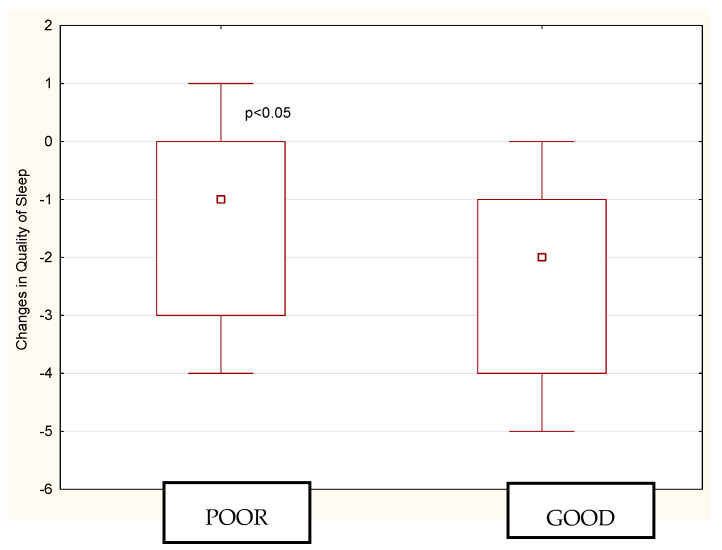
A subanalysis of sleep quality changes among poor sleepers vs. good sleepers (before Ramadan).

The PSQI results are presented in Table 2. In general, most of the athletes had low quality of sleep during both the period before Ramadan (BR) and during Ramadan (DR), although sleep quality decreased significantly DR (*p* < 0.001). The subjective sleep quality recorded DR was significantly poorer than that BR (*p* < 0.05). These results were consistent with those previously observed by Boukhris (2020) who also noticed a significant effect of Ramadan on sleep quality (*p* = 0.0006) (Table 3). In Herrera (2012) the values of subjective sleep quality recorded by PSQI were higher during Ramadan compared with those before Ramadan, but the differences were not statistically significant (Table 3). Compared to BR, a pair-wise comparison indicated that the sleep latency increased DR (*p* < 0.001) and daytime disfunctions intensified (*p* < 0.05). However, nonsignificant differences were noticed between the two time intervals for sleep duration, habitual sleep efficiency, sleep disturbances and use of sleeping medications (*p* > 0.05). A subanalysis of sleep quality changes among poor sleepers vs. good sleepers (before Ramadan) also showed significant differences (*p* < 0.05), meaning that among athletes who had good sleep quality previously, deterioration in sleep quality was much more noticeable (Figure 1).

### 3.3. Fitness

The results of fitness tests are presented in Table 4 and the changes in the results between BR and DR are presented in Table 5, Table 6 and Table 7. Generally, athletes obtained better physical fitness results (Cooper Test and Step Test) BR than DR, with statistically significant differences (*p* < 0.05).

The Cooper test showed that athletes, on average, covered 3585.09 ± 332.65 BR and 3521.56 ± 327.70 DR (*p* = 0.023) and that VO2max (mlO2/kg/min.) decreased from 68.86 ± 7.44 BR to 67.44 ± 7.33 BR (*p* = 0.023) (Table 4). The Step test BR showed that the resting heart rate of the athletes was 71.16 ± 12.06 and decreased to 68.47 ± 9.81 DR (*p* = 0.001). Furthermore, the peak heart rate was 97.59 ± 17.87 BR and increased to 102.84 ± 20.90 DR (*p* = 0.002) suggesting higher body fatigue (Table 4). These results are in line with those obtained by Roy (2015) showing a significant decrease in mean value of VO2max following Ramadan fasting, which indicated a significant reduction in aerobic capacity as well as in cardiorespiratory fitness. Analyzing the results of 6MWT from the study of Miladi (2020), it is also observed that the results were lower DR in comparison to BR, although the differences were not significant (Table 3). In the same study, the HR at rest and HR peak were also slightly higher DR in comparison to BR (Table 3).

### 3.4. Fitness vs. Sleep Quality

A positive association was observed between the quality of sleep and physical fitness (Cooper Test results). Athletes who were characterized by poor quality of sleep had lower physical fitness, both BR and DR. However, statistically significant differences were observed only in the results obtained BR (*p* < 0.05). DR, although physical fitness decreased with poor compared to good quality of sleep, the difference was not statistically significant (Table 5, Table 6 and Table 7).

### 3.5. Sleep Quality and Performance per Work Status

**Figure 2:**
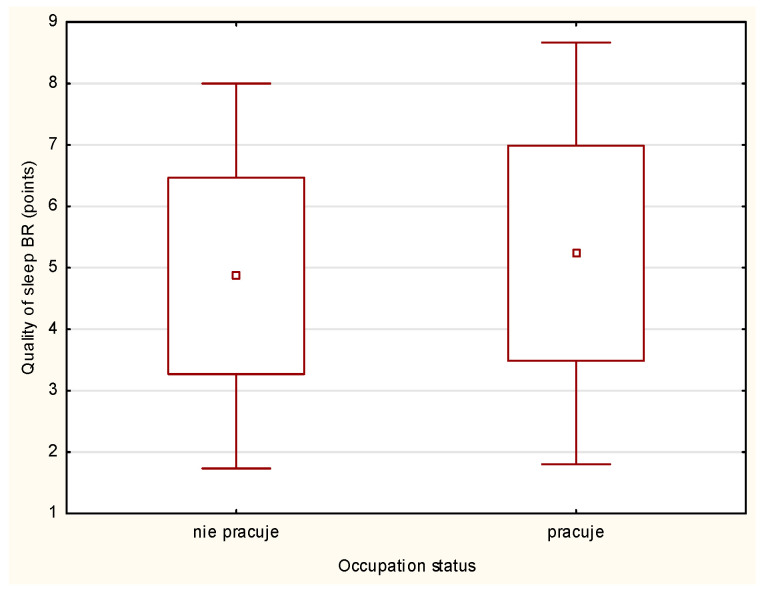
Quality of sleep according to the occupational status BR.

**Figure 3:**
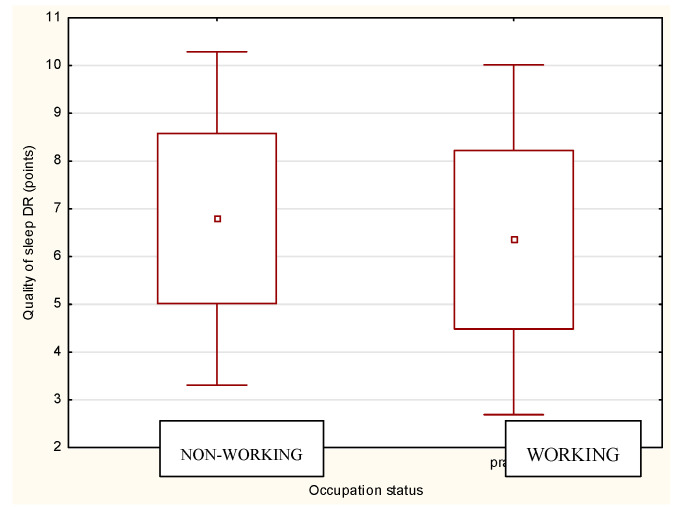
Quality of sleep according to the occupational status DR.

The analysis of physical performance and achieved quality of sleep according to work status is presented in Figure 2 and Figure 3. The nonworking athletes reported better quality of sleep than working athletes both BR and DR, but the differences were nonsignificant. The analysis of the physical performance of working and nonworking athletes BR and DR revealed nonsignificant differences between the groups (Table 5, Table 6 and Table 7). However, sleep quality achieved by working athletes was rated worse compared to that of nonworking athletes both during BR and DR. Sleep quality influenced physical performance, which was significantly lower compared to athletes who did not work (*p* < 0.05). This type of relationship was also observed in the DR period, although the differences were no longer statistically significant. Nevertheless, the physical performance of working athletes with good sleep quality was still higher compared to that of working athletes with poor sleep quality (Table 5, Table 6 and Table 7).

## 4. Discussion

The current study revealed that both quality of sleep and the physical performance of athletes deteriorated during Ramadan, but people with better quality of sleep had much better physical fitness/performance in both periods. It also showed that athletes who worked actively beside trainings achieved slightly worse physical fitness test results and had worse quality of sleep, although the differences were not statistically significant.

Sleep is an essential component of health and well-being, with significant impact on physical development [34]. Previous evidence suggests that increased sleep duration and improved sleep quality in athletes are associated with improved performance and competitive success [34], but further research is required to obtain better knowledge about the association between sleep and performance [35]. The current literature analysing this topic is sparse and much of the available information has been collected from sedentary subjects or low-level competitors. Existing evidence suggests that the impact of RDF on athletic performance is small relative to the precision of test procedures, but negative effects vary widely with the type of sport, the season when fasting is observed, the local culture and the discipline exercised by the athlete [36].

In general, most of the athletes had poor quality of sleep before or during Ramadan. Self-reported sleep duration, habitual sleep efficiency and sleep disturbances were the same before and during Ramadan. Unfortunately, sleep loss is a common occurrence in athletes. Most studies have found that the athletes failed to obtain the recommended amount of sleep because of training and competition schedules, travel, stress, academic demands and overtraining [34]. During Ramadan, the general quality of sleep decreased among the athletes, the sleep latency increased and daytime disfunctions intensified. Subjective sleep quality also decreased during Ramadan. This may be due to the fact that training, competition and eating are all concentrated into the hours of darkness, which causes chronobiological changes impairing sleep-wake patterns [17]. Moreover, changes in food intake, e.g., total energy, meal timing, meal composition or liquid intake, may also potentially affect physical performance. Food impacts the availability of tryptophan and the synthesis of serotonin and melatonin, which seems to be most helpful in promoting sleep. The physiological connections behind these effects are clear. Although, the evidence confirms a link between sleep and diet, the mechanisms of sleep are only partially clear and are the subject of intensive research [37]. Generally, previous studies that accounted for lifestyle factors and sleep/wake patterns have reported no changes in markers of the biological clock, daytime sleepiness or sleep parameters during diurnal intermittent fasting for Ramadan [38]. However, it could be interesting to obtain measurements of subjectively reported chronotype, or the ability to calculate some measure of daily sleep regularity among the runners, as in the study among Qatari football players who showed modest changes in chronotype, especially in the time preference for training, which was largely delayed during Ramadan and could influence sports performance [39].

Lack of sufficient sleep can significantly impact athletic performance, but the extent, influence, and mechanisms of sleep loss affecting exercise performance remain uncertain [35]. While previous studies showed that fasting had no effect on circadian rhythms [40], recent data suggest that fasting influences circadian rhythm [5].

Generally, participants presented worse physical performance during rather than before Ramadan. A previous study performed among soccer athletes showed that the phase shift of food intake and disruption of sleep patterns may affect actual and perceived physical performance [17]. Chennaoui et al. confirmed that sleep disturbances, energy deficiency and fatigue during Ramadan may decrease physical performance in Muslim athletes who maintain endurance training [21]. However, it was noticed that the physical performance was worse among athletes with poor, rather than with those with good quality of sleep. These differences were observed both in the pre-Ramadan and Ramadan period. Although previous studies investigating the effect of sleep loss on performance in athletes report a reduction in some sport-specific performance [35], findings remain conflicting in this topic [41,42].

Sleep quality was also important if the athlete was occupationally active. Athletes who worked and had poor sleep quality had significantly worse physical fitness, especially before the Ramadan month. It was also noticed that among athletes who reported good sleep quality, working athletes had slightly worse results in the Cooper test compared to nonworking athletes. It should be emphasized that apart from the load of the body resulting from fasting during Ramadan month, it may be important if the athlete works professionally or can only devote time to the trained discipline. Work can additionally burden the body, affect the quality of sleep and thus physical performance.

The main strength of this study is the sample type. According to our knowledge, no professional runners have been analysed so far. Although the study had a quasiexperimental design with an absence of a control group, participants were in their natural environment and generally used to fasting during Ramadan, so there was no need to match people artificially to the experiment. Next, the questionnaires and fitness tests selected for use in the study were international and well-standardized [43,44,45]. Moreover, sleep assessment has been never done before during fasting, or only selected sleep elements were analysed e.g., total sleep time or daytime sleepiness [11]. To our knowledge, no previous study analysed the influence of Ramadan month on physical performance in relation to the occupational status of the athlete.

However, some limitations should be mentioned. First, the sample size was homogenous, as only men training in one specific type of physical activity such as endurance-training were analysed. Thus, the results cannot be generalized to the whole group of professional athletes participating in fasting in the month of Ramadan. Moreover, the results cannot be generalized to female athletes, as potentially different associations could be observed since sleep characteristics could vary by sex. Second, the study was limited by the small sample size and requires more extensive research that would confirm the obtained results. Extensive food intake control with the adherence to eating suhur and/or the timing of suhur would be recommended to compare results based on eating behaviours.

It could be interesting to explore the changes in physical performance and sleep during every week of Ramadan. However, based on data collection time in a systematic review about the issue of fasting and performance in athletes [12], and our own research [10,30,31], we noticed that only few studies have measured performance in the four weeks of Ramadan, and the majority of the studies made measurements only in one week (usually the 3rd or 4th). For this reason, and for logistic complications in data collection at several time intervals, we decided to collect data before and at one time interval during (3rd week) Ramadan. Moreover, there was no typical control group, but the study was performed in the natural environment of the religiously practicing athletes, so it was impossible to require that some athletes not adhere to Ramadan fasting. In order to solve this problem, we decided to refer to the results of other studies conducted on a similar subject and on a similar group of people as our reference point (Table 4). It would also be interesting to discuss the role of the physical activity, besides the role of fasting, on sleep patterns. Although we analyzed the changes in the amount of training undertaken before and during Ramadan, future research gathering data about physical activity before and during Ramadan as a possible confounding variables would be recommended.

Results should be treated with caution with respect to their interpretation and comparison with previous studies. As daytime length (time of abstinence from food and drinks) and atmospheric temperature during Ramadan differ among countries, it is important to take into consideration the country of the study upon comparing evidence from research s about Ramadan fasting and physical performance. Besides, while a substantial amount of literature has explored the effect of intermittent fasting on physical performance [12], it important to note that RDF is different. RDF means neither eating nor drinking any kind of fluid, while intermittent fasting allows drinking noncaloric drinks such as coffee and tea.

Implications for future research:

It would have been interesting to discuss the role of the physical activity, besides the role of fasting, on sleep patterns. In our study, we aimed to study the role of fasting in the month of Ramadan on physical performance and sleep quality However, by fasting in the month of Ramadan, we did not mean to study the role of abstaining from food and nutrients per se, but also the role of all that fasting involves in changes in eating habits, metabolic rhythm and disturbed circadian rhythm, as well as daily habits and physical activity. However, the physical activity, as a possible confounding variable in the association between fasting and performance, was not measured in the questionnaire. We mention this limitation in the discussion section and point it out to be an implication for future research when gathering data about physical activity before and during Ramadan in order to control for physical activity as a possible confounding variable.

## 5. Conclusions

In light of results of this study, athletes may require more careful monitoring during RDF to promote proper sleep patterns and to improve sleep quality and physical performance. Special strategies should target athletes who have job schedules besides training, to take into account the effect of work on training and performance. These might include evaluating the benefit of changing the training time schedule or training during weekends.

## Figures and Tables

**Table 1:** Anthropological and training characteristics of the study participants (n = 32).

Age (years)		28.28 ± 6.67
Body weight (kg)		69.58 ± 8.18
Body height (cm)		174.59 ± 7.01
Years in sport		10.19 ± 4.94
Smoking (fraction of people)	Manual work	0.44
	Office work/ student	0.56
Working (fraction of people)	Yes	0.53
	No	0.47
Training characteristics before Ramadan		
No. training hours (hours/week)		10.59 ± 2.79
No. trainings per day (fraction of people)	Once a day	0.56
	Twice a day	0.44
Training characteristics during Ramadan		
No. training hours (hours/week)		8.03 ± 1.66
No. trainings per day (fraction of people)	Once a day	0.97
	Twice a day	0.03

**Table 2:** Subjective sleep quality (mean ± SD) as estimated by the Pittsburgh Sleep Quality Index (PSQI). Values before (BR) and during (DR) Ramadan.

	BR	DR
Quality of sleep (Global PSQI score) (AU)	5.06 ± 1.66	6.56 ± 1.81 *
Component 1: subjective sleep quality (AU)	0.97 ± 0.74	1.59 ± 0.91 *
Component 2: sleep latency (AU)	0.81 ± 0.54	1.09 ± 0.59 *
Component 3: sleep duration (AU)	1.03 ± 1.09	1.03 ± 1.09
Component 4: habitual sleep efficiency (AU)	0.0	0.0
Component 5: sleep disturbances (AU)	1.00 ± 0.25	1.03 ± 0.18
Component 6: use of sleeping medications (AU)	0.25 ± 0.57	0.25 ± 0.57
Component 7: daytime dysfunction over the last month (AU)	1.00 ± 0.44	1.56 ± 0.72 *

*: Significant difference compared to BR. AU: arbitrary units.

**Table 3:** Results of previous studies concerning the influence of Ramadan fasting on physical performance and quality of sleep.

	Subjects	Measured Parameter	Tests	Main Results		
				BR	DR	Conclusion
Hsouna H. (2020) [29]	14 physically active males; 7 with 35-min nap (N35) and 7 without nap (N0)	Sleep quality	PSQI	N0 3.86 ± 0.47 N35 3.43 ± 0.31	N0 4.50 ± 0.45 N35 4.07 ± 0.40	There was a significant increase in subjective sleep quality scores DR in comparison with BR.
Boukhris O. (2019) [30]	14 physically active Arabic men	Sleep quality	PSQI	3.3 ± 2.3	6.7 ± 2.6	The sleep quality score was higher during Ramadan in comparison to before Ramadan.
Herrera C. (2012) [10]	14 Muslim adult male football players	Sleep quality	PSQI	5.3 ± 3	5.8 ± 3	Although the differences were not statistically significant, the values during Ramadan were higher compared with before Ramadan.
Güvenç A. (2011) [31]	16 soccer players	Aerobic physical performance	Shuttle Run Test	RPE 14.75 ± 1.48	RPE 16.00 ± 1.75; 15.69 ± 1.30	There was an increase in subjective ratings of perceived exertion at submaximal intensities during Ramadan.
				70.24 ± 10.56 HR at rest	68.59 ± 9.14; 67.53 ± 9.91 HR at rest	
				192.78 ± 7.32 HR	193.49 ± 7.97; 191.98 ± 7.19 HR	
Miladi A. (2020) [32]	34 boys; 26 fasters and 10 non-fasters	Cardiorespiratory capacity	6MWT	F 680 ± 76 NF 635 ± 60	F 679 ± 98 NF 619 ± 69	RO did not impact the 6MWD HR value.
				F 78 ± 7 HR at rest NF 79 ± 6 HR at rest	F 79 ± 9 HR at rest NF 80 ± 7 HR at rest	
				F 122 ± 14 HR response NF 118 ± 13 HR response	F 120 ± 21 NF 118 ± 18	
Roy AS. (2015) [33]	77 young untrained Muslim men; 40 control group (CG) and 37 experimental group (EG)	Aerobic physical performance	Sumaximal test on ergometer (VO2max measurement)	CG 43.73 ± 5.13 mL/kg/min EG 44.02 ± 5.24 mL/kg/min	CG 44.96 ± 4.84 mL/kg/min EG 39.23 ± 4.41 mL/kg/min	VO2max values in EG were significantly lower than those in CG during the month of Ramadan

**Table 4:** Physical performance (mean ± SD) as estimated by the Cooper test and Step test. Values before (BR) and during (DR) Ramadan.

		BR	DR	t	*p*
Cooper test	Covered distance (meters)	3585.09 ± 332.65	3521.56 ± 327.70	2.398	0.023
	VO2max. (mlO2/kg/min.)	68.86 ± 7.44	67.44 ± 7.33	2.398	0.023
Step test	Heart rate at rest (per min)	71.16 ± 12.06	68.47 ± 9.81	3.441	0.001
	Heart rate after the step test (per min)	97.59 ± 17.87	102.84 ± 20.90	3.308	0.002

*p* < 0.05 was statistically significant.

**Table 5:** Physical performance (mean ± SD) as estimated by the Cooper test according to the occupational status and quality of sleep. Values before (BR) and during (DR) Ramadan.

VO2max. in Cooper Test (mlO2/kg/min)	BR	DR	t	*p*
	Mean (±SD)			
Occupational status				
Working	68.20 ± 8.60	66.97 ± 8.18	1.487	0.156
Nonworking	69.62 ± 6.06	67.96 ± 6.47	1.864	0.083
Quality of sleep				
Poor	65.82 ± 6.92	64.94 ± 7.07	1.071	0.297
Good	74.66 ± 4.37	72.21 ± 5.35	3.795	0.003

**Table 6:** Heart Rate at rest of the study participants according to the occupational status and quality of sleep. Values before (BR) and during (DR) Ramadan.

HR at Rest	BR	DR	t	*p*
	Mean (±SD)			
Occupational status				
Working	71.12 ± 12.56	67.65 ± 10.58	3.041	0.008
Nonworking	71.20 ± 11.90	69.40 ± 9.15	1.726	0.106
Quality of sleep				
Poor	70.43 ± 12.85	67.62 ± 9.72	2.572	0.018
Good	72.54 ± 10.82	70.09 ± 10.26	2.539	0.029

**Table 7:** Physical performance (mean ± SD) as estimated by the Step test according to the occupational status and quality of sleep. Values before (BR) and during (DR) Ramadan.

HR Peak in Step Test	BR	DR	t	*p*
	Mean (±SD)			
Occupational status				
Working	96.18 ± 15.66	101.12 ± 18.66	−2.602	0.019
Nonworking	99.20 ± 20.54	104.80 ± 23.70	−2.086	0.056
Quality of sleep				
Poor	97.52 ± 20.41	102.24 ± 24.09	−2.608	0.017
Good	97.73 ± 12.51	104.00 ± 13.84	−1.976	0.076

## Data Availability

The data presented in this study are available on request from the corresponding author.

## Correction Statement

The Editorial Office and Editorial Board would like to inform readers of a post-publication review process that was conducted related to this publication. Following concerns about a potential conflict of interest between the authors and the original Academic Editor, the Editorial Office has conducted a post-publication peer-review of this article. This process included the recruitment of a new Academic Editor to ensure full compliance with MDPI’s Editorial Process (https://www.mdpi.com/editorial_process). As a result of this process, the original Academic Editor was removed, and a new Academic Editor is listed. The new Academic Editor is satisfied that the editorial process for this article has been completed in accordance with MDPI’s Editorial Process policy.

## Abbreviations

RDF	Ramadan Diurnal Fasting
PSQI	Pittsburgh Sleep Quality Index
BR	Before Ramadan
DR	During Ramadan
